# Application of an adjusted patient blood management protocol in patients undergoing elective total hip arthroplasty: towards a zero-percent transfusion rate in renal patients—results from an observational cohort study

**DOI:** 10.1186/s13018-021-02846-z

**Published:** 2021-11-27

**Authors:** Hervé Hourlier, Peter Fennema

**Affiliations:** 1 Department of Orthopaedic Surgery, Polyclinique de la Thiérache, Rue du Dr Koral, 59212 Wignehies, France; 2 AMR Advanced Medical Research, Männedorf, Switzerland

**Keywords:** Patient blood management, Tranexamic acid, Total hip arthroplasty, Blood transfusion, Chronic kidney disease

## Abstract

**Background:**

Renal patients are at high risk of blood transfusion following major orthopaedic surgery. A variety of patient blood management (PBM) policies have been proposed to reduce the rate of transfusions. The aim of this observational study was to assess the performance of an adjusted PBM protocol in patients with chronic kidney disease (CKD) undergoing elective total hip arthroplasty (THA).

**Methods:**

A total of 1191 consecutive patients underwent elective unilateral THA and took part in an adjusted PBM protocol. The PBM protocol consisted of epoetin (EPO) alfa therapy prescribed by the surgeon, routine administration of tranexamic acid (TXA), an avascular approach to the hip and postoperative prophylaxis of thromboembolism. The performance of this PBM protocol was analysed in patients with a glomerular filtration rate (GFR) below or above 60 ml/min/1.73 m^2^ at baseline. Haemoglobin levels were controlled at admission, on postoperative day (POD) 1 and on POD 7 ± 1. A bleeding index (BI) was used as a proxy for blood loss.

**Results:**

In total, 153 patients (12.9%) presented with a modification of diet in renal disease value below 60 at baseline. Of these, 20 (13.1%) received EPO therapy and 120 (78.4%) received TXA. None of the patients received allogenic blood transfusions during the first perioperative week. The mean BI for the entire study population was 2.7 (95% CI 2.6, 2.8). CKD did not exert a significant impact on the BI (*p* = 0.287). However, it was found that both TXA and EPO therapy significantly lowered the BI (difference, − 0.3, *p* < 0.001). There were no thromboembolic complications in renal patients who received TXA and/or EPO therapy.

**Conclusions:**

A zero-percent transfusion rate during the first perioperative week is attainable in patients with stage 3 or stage 4 CKD undergoing contemporary elective THA. With the use of a pragmatic blood-sparing protocol, patients with renal dysfunction did not have an increased risk of bleeding and did not have an increased incidence in the rate of perioperative blood transfusions.

## Background

Total hip arthroplasty (THA) is the treatment of choice for patients with advanced osteoarthritis of the hip [1]. Demographic changes will increase the future demand for THA procedures [2]. Chronic kidney disease (CKD) has become much more prevalent [3], particularly in the older population. Its prevalence has been estimated at 9.1% of the population, and the global all-age prevalence of CKD has increased 29.3% since 1990 [4]. Previous studies have shown that following THA surgery, the presence of CKD is an independent risk factor of postoperative transfusion, bleeding complications, 30-day readmission, thrombosis and mortality [5–12]. Transfusions following THA surgery are frequent in patients with CKD, because renal impairment is often accompanied by preoperative anaemia and cardiovascular comorbidities, which bring postoperative haemoglobin (Hb) levels closer to the transfusion trigger [10].

Regarding total joint arthroplasty (TJA), transfusion during total knee and hip arthroplasty is associated with the risk of surgical site infection, major complications, longer hospital stays and even mortality [11, 13, 14]. Furthermore, transfusion increases the costs of hospitalisation and resource utilisation [12, 14]. Consequently, there is an ongoing need to further reduce blood loss.

A variety of patient blood management (PBM) protocols have been proposed to reduce the rate of blood transfusions in orthopaedic surgery [15]. PBM is an evidence-based-care bundle with a proven ability to improve patient outcomes by managing and preserving the patient’s own blood. Since 2010, the World Health Organization has called on member states to adopt PBM protocols [16]. Unfortunately, there has been limited progress in developing PBM programs in hospitals due to the implicit challenges of implementing them [16].

In 2005, we initiated a PBM protocol in our unit; the protocol included the use of drugs instead of autotransfusion [17, 18]. Tranexamic acid (TXA) was administered routinely, and epoetin (EPO) alfa therapy was prescribed in accordance with the product’s instructions for use (a standardised regimen of one weekly 40,000-IU EPO alfa injection for four weeks) and the national guidelines’ indications (preoperative Hb between 10 and 13 g/dl). In 2011, we reaudited our PBM plan and decided to implement some changes to maximise the cost-effectiveness of EPO therapy [19]. That is, EPO therapy was prescribed by the surgeon, the upper preoperative Hb threshold was lowered to 12 g/dl and the number of injections before surgery was reduced to two [19]. Routine prescription of oral iron supplementation (i.e. even in the absence of a specific deficiency) was abandoned. The aim of this adjusted PBM protocol was to achieve an efficient, easily applicable, cheaper and low-risk way to eliminate allogenic blood transfusions and reduce the severity of postoperative anaemia [19]. We now report on the effectiveness of this adjusted PBM protocol.

We aimed to evaluate whether this PBM was effective in patients undergoing contemporary THA with kidney problems at baseline. Renal patients undergoing THA are typically elderly people and regular users of antithrombotic medications, which expose them to excessive bleeding [20].

The second aim of this study was to evaluate the respective contributions of TXA and EPO therapy to the final results. We therefore assessed whether TXA and preoperative EPO therapy led to a similar reduction in postoperative Hb levels and to a similar incidence of allogenic blood transfusions in CKD patients. We hypothesised that with the use of our adjusted PBM protocol, a small difference in blood loss between patients with and those without CKD would be found.

## Methods

A retrospective study using prospectively collected data was conducted in a cohort of unilateral primary elective THAs performed by a single surgeon from January 2011 to December 2019. The exclusion criteria were revision arthroplasties, bilateral THA procedures and acute fracture procedures. Patients who received therapeutic-dose heparin or underwent renal dialysis before surgery were also excluded. In total, 1191 consecutive patients were included in the study. Participants were dichotomised as presenting with CKD when they had a preoperative glomerular filtration rate (GFR) below 60 mL/min/1.73 m^2^ [21], calculated with the modification of diet in renal disease (MDRD) equation [22]. All patients took part in the PBM protocol, which comprised preoperative use of EPO therapy if indicated, use of TXA, thromboembolism prophylaxis and contemporary blood-sparing surgical techniques [17, 19].

### Preoperative PBM protocol

As per our routine protocol, within a three-month period before the preoperative surgical consultation, an initial blood sample was drawn in all patients scheduled for THA. During the preoperative surgical consultation, conducted one month before surgery, anaemic patients were identified. If baseline Hb levels were 10–12 g/dL, preoperative Hb optimisation consisted of the surgeon’s prescription of two injections of a subcutaneous dose of EPO once a week for two weeks before the surgery [19]. Contraindications to EPO therapy were allergy to EPO, history of erythroblastopenia and uncontrolled arterial hypertension. EPO therapy was combined with oral supplementation of iron.

Patients who received anticoagulation medication before the surgery were invited to temporarily discontinue their antithrombotic therapy. The day before the surgery, a blood count was performed in all the patients.

### Intraoperative PBM protocol

For all patients, a less invasive anterolateral approach in a lateral position, which provides good exposure of the acetabulum and proximal femur, was used to pose a cementless prosthesis [23]. No vessel ligation was required. The capsule was repaired to obliterate the dead spaces. All the procedures were performed under general anaesthesia preceded by ultrasound-guided blocking of the femoral nerves. Hydration was low. No surgical drain or blood salvage system was used. No analgesic local infiltration was administered by the surgeon, because all the patients underwent ultrasound-guided nerve blocks. Standard antibiotic regimens were administered 30–60 min before the skin incision.

Intraoperatively, a single-shot IV of 20–30 mg/kg TXA was routinely administered via a minibag of saline before the skin incision [24].

TXA was not administered in patients with a history of venous or arterial thromboembolic events within the previous three months. Moreover, patients with erythrocytemia were not given TXA, because they were not considered to be at risk of postoperative blood transfusion. Based on these criteria, 179 patients (15.0%) did not receive TXA. Notably, the cohort included a patient who did not receive TXA because of a previous renal replacement.

### Postoperative PBM protocol

Six to eight hours after closure, 10 mg oral rivaroxaban (Xarelto; Bayer, Lille, France) was given to initiate pharmacological venous thromboembolism prophylaxis, which was prolonged for 35 days. No other anticoagulants were administered [25].

Multimodal pain management was applied during the postoperative period. In patients without comorbidities, blood transfusion was administered at an Hb threshold of 7 g/dl; in patients with anaemia symptoms, pre-existing cardiac insufficiency or coronary heart disease, it was administered at a threshold of 8 g/dl.

### Precautions in renal and cardiac patients

In patients considered to be at risk of cardiovascular complications, monitoring for troponin took place postoperatively. Immediate postoperative diuresis volume was quantified in all the patients. Bladder scans were used routinely to detect urinary retention.

Nephrotoxic medications were avoided during the postoperative course. Specifically, nonsteroidal anti-inflammatory drugs were used sparingly in all patients.

### Outcomes

In this study, Hb levels were determined only using venous blood samples, and they were controlled by the same laboratory at admission (postoperative day [POD] -1), on POD 1 and on POD 7 ± 1. For patients discharged before POD 6, blood was collected at home on POD 7 to control Hb. Any blood transfusions were recorded.

A bleeding index (BI) was the primary endpoint and was calculated for each patient as follows: Hb level (g/dL) on the day before surgery minus Hb level on POD 7 plus the number of units of packed red blood cells (PRBCs) transfused between those two time points, based on the assumption that the transfusion of one unit of PBRCs increases Hb by 1 g/dl [26, 27].

Females with Hb under 12 g/dl and males with Hb under 13 g/dl were considered anaemic [28].

The recording of all complications was carried out for 90 days. The following events were considered major complications: death, perioperative myocardial infarction, cerebrovascular accident, proximal deep venous thrombosis (DVT), infection and symptomatic pulmonary embolism (PE). Blood transfusion was the secondary study endpoint.

Written informed consent was obtained from all the patients prior to study commencement. None of the patients enrolled in the study reported having religious convictions against blood transfusion. Ethics committee approval was not sought, because the study was purely observational and resulted in no changes to standard clinical practice; this is in accordance with French law.

For continuous variables, baseline data are presented as mean ± standard deviation (SD); for categorical variables, baseline data are presented as counts and percentages. Multiple linear regression was used to test the primary research question. We adjusted for the confounders of age, sex, body mass index (BMI), surgical time, preoperative use of EPO therapy and baseline Hb level, which were considered clinically relevant. *P* values under 0.05 indicated statistical significance. Stata 15.1 (Stata Corp, College Station, TX, USA) software was used for statistical analysis.

## Results

In total, 1191 patients were included in the study. Of these, 153 (12.9%) presented with CKD. The baseline characteristics of the study population are presented in Table 1. In the CKD group, 20 patients (13.1%) received preoperative EPO therapy and 120 (78.4%) received intraoperative TXA. None of the renal patients underwent transfusion in the interval between the day before surgery and the blood sample, taken 6–7 days postoperatively for Hb determination. Within the first three months after surgery, two patients (1.3%) with CKD and one patient (0.1%) without CKD died from unrelated causes (*p* = 0.045). During the first postoperative week, no patients received allogenic blood transfusions. The mean BI for the entire study population was 2.7 (95% CI 2.6, 2.8). The BI difference between CKD and non-CKD patients was statistically nonsignificant (difference, 0.1 [95% CI − 0.1, 0.2], *p* = 0.543). Patients who received TXA had a significantly lower BI (difference, − 0.3 [95% CI − 0.5, − 0.1], *p* < 0.001).

**Table 1:** Baseline characteristics

	CKD- (*n* = 1038)	CKD + (*n* = 153)	*p* value
Preoperative GFR (mL/min/1.73 m^2^)^§^	91.9 ± 21.8	49.2 ± 8.6	–
Stage 1 (GFR: ≥ 90)	490 (47.2%)	0 (0.0%)	–
Stage 2 (GFR: 60–89 mL/min/1.73 m^2^)	548 (52.8%)	0 (0.0%)	
Stage 3a (GFR: 45–59 mL/min/1.73 m^2^)	0 (0.0%)	111 (72.6%)	
Stage 3b (GFR: 30–44 mL/min/1.73 m^2^)	0 (0.0%)	37 (24.2%)	
Stage 4 (GFR: 15–29 mL/min/1.73 m^2^)	0 (0.0%)	5 (3.3%)	
Stage 5 (GFR: < 15 mL/min/1.73 m^2^)	0 (0.0%)	0 (0.0%)	
Age (years)	69.3 ± 10.1	77.1 ± 10.1	< 0.001
Sex (female)^§^	504 (48.7%)	98 (64.1%)	< 0.001
BMI (kg/m^2^)	29.2 ± 5.3	29.1 ± 5.6	0.795
Hb at the consultation (g/dL)	13.9 ± 1.3	13.1 ± 1.2	< 0.001
Anaemic at the consultation^§^	106 (10.1%)	29 (19.0%)	0.001
Hb the day before surgery (g/dl)	14.1 ± 1.3	13.1 ± 1.4	< 0.001
Anaemic the day before surgery^§^	124 (11.7%)	29 (21.5%)	< 0.001
Antithrombotic drug regular use^§^	330 (31.8%)	80 (52.3%)	< 0.001
Preoperative EPO therapy^§^	27 (2.6%)	20 (13.1%)	< 0.001
TXA use^§^	892 (85.9%)	120 (78.4%)	0.015
Dose (mg/kg)	24.5 ± 5.7	24.3 ± 5.1	0.803
ASA^§^			< 0.001
ASA 1	223 (22.6%)	3 (2.0%)	
ASA 2	678 (65.8%)	101 (66.9%)	
ASA 3	119 (11.6%)	47 (31.1%)	
Surgical time (min)	59.7 ± 10.1	57.8 ± 12.1	0.042

Presented as mean ± standard deviation, except ^§^, presented as number of observations (%). Abbreviations: ASA, American Society of Anesthesiology; CKD, chronic kidney disease; EPO, erythropoietin; GFR, glomerular filtration rate; TXA, tranexamic acid

The BI reduction was also significant in patients who received EPO therapy (difference, − 0.4 [95% CI − 0.7, − 0.1, *p* = 0.008]) (Table 2). None of the CKD patients who received TXA experienced a proximal DVT or a PE. In the CKD group, one patient presented renal function degradation due to urosepsis, which was treated with antibiotics. No other major complications occurred. No patient underwent reoperation during the first postoperative week.

**Table 2:** Results

	CKD- (*n* = 1038)	CKD + (*n* = 153)	*p* value
BI	2.7 (2.7–2.8)	2.8 (2.6–2.9)	0.543
RBC transfusion between day before surgery and POD 7 ^§^	0 (0.0%)	0 (0.0%)	1.00
Hb on POD 7	11.1 (11.1–11.2)	10.8 (10.6–11.0)	0.006
Anaemic on POD 7 ^§^	983 (94.7%)	147 (96.1%)	0.471
GFR (mL/min/1.73 m^2^) on POD 7	95.3 (93.7–96.9)	53.8 (53.1–54.4)	< 0.001

Presented as mean (95% confidence interval), except ^§^, presented as number of observations (%). Abbreviations: CKD, chronic kidney disease; GFR, glomerular filtration rate; EPO, erythropoietin; POD, postoperative day; RBC, red blood cell

## Discussion

Reducing the use of allogenic blood is an important means of improving outcomes in terms of both mitigating risks to patients and lowering costs in healthcare systems [29, 30].

As a recent study demonstrated, patients with kidney problems are at the highest risk of undergoing transfusion following TJA surgery [6]. The present study shows that the use of our adjusted PBM protocol was associated with a zero-percent transfusion rate as well as acceptable postoperative Hb concentrations in a large series of consecutive patients, including patients with stage 3 or stage 4 CKD. No significant BI difference between CKD and non-CKD patients was found.

In the current series, the PBM protocol was driven by a close collaboration between a senior surgeon and a senior anaesthetist. Before the operation, the surgeon identified patients with risk factors for transfusion, such as advanced age, female gender, a baseline Hb less than 12 g/dL, cardiac disease, receiving antithrombotic drugs, lower-than-average body weight or prolonged duration of surgery due to technical challenges. The date of surgery was scheduled in accordance with the baseline Hb level. The surgeon prescribed EPO therapy in patients with anaemia at high risk of undergoing transfusion.

In the current study, the adjusted PBM protocol consisted of four main elements: preoperative Hb optimisation, the use of TXA, management of antithrombotic drugs and blood-sparing surgical techniques [17, 19].

### Epoetin alfa

The use of EPO therapy is an important part of our PBM protocol. The increased erythropoiesis that occurs early postoperatively has a direct effect on blood loss [31]. Furthermore, the increase in the preoperative Hb reduces the rate of postoperative anaemia and the number of patients who reach the transfusion threshold [19]. However, the BI found in the present study combined with the use of a conservative transfusion policy suggests that the Hb concentration of 13 g/dl, which is currently recommended for preoperative EPO-therapy administration, was too high in our practice.

The main limitation of preoperative EPO therapy remains its cost. However, the economic cost of EPO therapy must be balanced against the costs of blood transfusion and against the health and economic consequences of complications associated with postoperative anaemia. Because the cost and safety of EPO therapy are dose dependent, we believe our model is economical and safe; we used both a lower Hb threshold and a lower dose for EPO therapy than reported elsewhere [32].

In the present study, more CKD than non-CKD patients were treated with EPO therapy because the proportion of preoperative anaemia is higher in renal patients than in patients without renal problems.

Erythropoietin hormone is produced by the kidneys, and its production is reduced in renal patients. Therefore, EPO recombinant therapy is considered more efficient in patients with kidney problems.

Importantly, EPO therapy is safer when administered with a low dose. In the current series, EPO therapy was prescribed by the surgeon sparingly. No patients received EPO therapy or iron during the immediate postoperative period. Despite the prescription of a lower-than-average dose of EPO, it was found that EPO therapy effectively reduced the BI in CKD patients.

### Tranexamic acid

With the administration of TXA and the advances in surgical techniques over the last two decades, the historical postoperative Hb drift of 4.1 g/dl (with a standard deviation of 1 g/dl) associated with THA [33] has been reduced to less than 3 g/dl in contemporary THA.

In the present series, most of the renal patients received TXA. A recent registry study demonstrated the efficacy of TXA in TJA patients with renal impairment [9]. TXA reduced the transfusion rate from 23.3 to 9.1% in patients with renal impairment. Importantly, this registry study—which included 765,011 patients, of whom 44,506 had renal impairment – reported no increase in the rate of complications among patients who received TXA, including the high-risk patients [9, 12].

In the present series, TXA was administered before the skin incision via a single infusion in patients at risk of undergoing transfusion. TXA was not associated with adverse effects. We used a single shot sufficiently dosed to inhibit fibrinolysis during the period of maximum blood loss. No repeated-dose regimen was administered. TXA is primarily eliminated via the kidneys, with over 95% excreted unchanged in urine [33]. The elimination of TXA is reduced in patients with reduced GFR [34] and/or low immediate postoperative diuresis. The use of a single shot prevents drug accumulation in renal patients. Several trials have found that single-shot TXA was as effective as a repeated-dose regimen [24, 35, 36].

### Strength and limitations

One of the present study’s strengths is that all data were collected prospectively, within a regular clinical practice setting. Furthermore, the study was homogeneous in terms of surgical technique and preoperative- and postoperative-care protocols. A limitation of the study is that it included few patients with stage 4 CKD. It has been reported that outcomes among CKD patients are not uniform and that patients may be at increased risk of bleeding, implant failure and postoperative infection as the disease progresses [37].

Observational studies carry the risk of confounding by unmeasured and/or unknown factors, which is another limitation of this study. Moreover, the present study’s CKD sample was too small to enable a determination of any differences in the rate of adverse drug reactions. Further, given the monocentric nature of the current study, the reproducibility of our results needs to be confirmed by other studies. Our study’s results are likely to be confounded by surgeon training and experience, the lack of reoperations and the preventive measures taken to prevent patients from falling during the hospital stay. All these factors contributed to reducing the risk of transfusion.

## Conclusions

A zero-percent transfusion rate after THA can be a realistic goal even in patients at high risk of bleeding. With the use of a pragmatic adjusted PBM protocol, patients with renal impairment had neither an increased risk of bleeding nor an increased incidence in the rate of perioperative blood transfusions.

## Data Availability

The datasets used and/or analysed during the present study are available from the corresponding author upon reasonable request.
